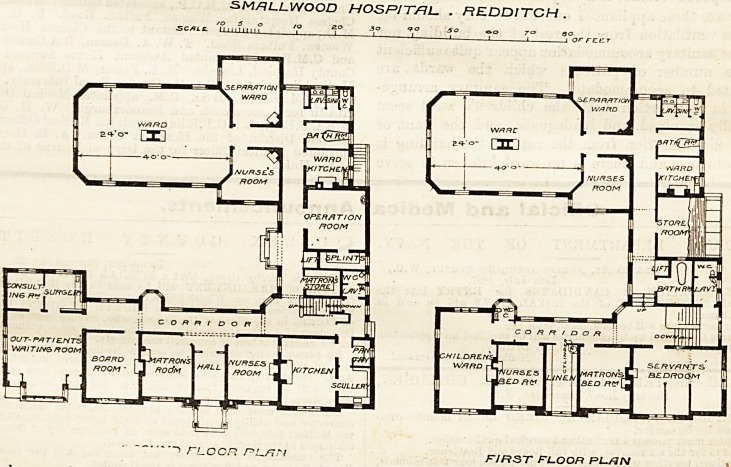# Hospital Construction

**Published:** 1897-10-02

**Authors:** 


### HOSPITAL CONSTRUCTION.

### THE SMALLWOOD HOSPITAL, REDDITCH.

This building is arranged on two floors round three sides of a quadrangle, the open side facing south. The laundry and mortuary buildings form a separate block on the west side, and stand at some distance from the hospital proper.

The wards and their appurtenances,—nurses' rooms, ward-kitchens, and lavatories occupy the western wing. The accommodation for in-patients—which is identical on the ground and first floors—is intended for male and female patients respectively, and consists of a large ward 40 feet by 24 feet for eight beds, a separation ward for two beds, a nurses' room adjoining the large ward and controlling it, and a bath-room, ward-kitchen, and sanitary accommodation.

SMALLWOOD HOSPITAL . REDDITCH.

[illegible] FLOOR PLAN

FIRST FLOOR PLAN

In the eastern wing, the ground floor is devoted to the administrative portion of the building, and provides a board-room, matron's-room, and nurses'-room close to the principal entrance. The out-patient department forms a one-storied building at the south end of this wing, consisting of a waiting-room, with consulting-room and surgery in connection with it.

On the first floor of the eastern wing, the matron's bed-room and a bed-room for a nurse are arranged over the nurses'-room and matron's-room on the ground floor, and a well-planned and cheerful children's ward stands across the southern end of the wing over the board-room.

The north wing provides the kitchen, scullery, &c., in the north-east angle on the ground floor, and an operating-room, with surgical and matron's store-rooms in the portion joining the eastern and western wings. Over these on the first floor are the bath-room and sanitary accommodation for the staff, and a servants' bed-room over the kitchen.

The principal staircase is at the angle of the corridors connecting the eastern and northern wings, and near it a lift rises from the basement through each floor. The basement in question extends under a part of the north wing, is approached by a staircase from the kitchen department working under the main stairs, and contains coal stores and larder, and the heating chamber, with its fuel store, which is approached directly from the outside.

It is to be observed in these plans that the children's ward has no cross lighting or ventilation; the general arrangement of the large wards seems good, and the plan affords them a maximum of sunshine, but the severance of the sanitary appliances from them by the interposed corridor is a very awkward arrangement; neither are these appliances cut off, as they should be, by cross ventilation from the rest of the building, nor does the sanitary accommodation appear quite sufficient for the number of patients which the wards are calculated to accommodate. The sanitary arrangements in connection with the children's ward seem especially cramped and inadequate, and the want of proper disconnection from the rest of the building is again obvious, and there is no ward kitchen to serve this ward, which would certainly require it not less than the others. There does not appear to be sufficient wall space in the separation wards to arrange the two beds comfortably which they are supposed to accommodate.

The operation-room is not convenient for patients on the first floor, as the lift is not shown sufficiently large to contain a stretcher for their conveyance.

In the out-patients' department the entire lack of any sanitary accommodation is surely a mistake, and the want of a dispensary in an institution of this size will almost certainly be felt. Another apparent oversight is the absence of any room in the whole building for the use of the medical practitioners connected with it.

The arrangement of the building on the site has evidently been made with great care as regards aspect, and reflects great credit on the architect, Mr. Henman, of Birmingham.

## Figures and Tables

**Figure f1:**